# Navigating Uncharted Waters: Steering the Course and Confronting Challenges of Brain Metastases in EGFR-Mutant Non-Small Cell Lung Cancer

**DOI:** 10.3390/cancers18162647

**Published:** 2026-08-17

**Authors:** Daniela Garcez, Ana Rodrigues, Catarina Travancinha, Marcos Pantarotto, Paulo Costa, António Araújo, Juan Rachadell, João Ramalho-Carvalho, Ana Figueiredo, Telma Sequeira, Maria Gabriela O. Fernandes

**Affiliations:** 1 Unidade de Tumores Cerebrais, CUF Oncologia, Hospital CUF Descobertas, 1998-018 Lisbon, Portugal; 2 Unidade de Tumores Cerebrais, CUF Oncologia, Hospital CUF Tejo, 1350-352 Lisbon, Portugal; 3 Department of Medical Oncology, Instituto Português de Oncologia do Porto, 4200-162 Porto, Portugal; 4 Unidade de Cancro do Pulmão, CUF Oncologia, Hospital CUF Tejo, 1350-352 Lisbon, Portugal; 5 Lung Unit Oncology, Fundação Champalimaud, 1400-038 Lisbon, Portugal; 6 Department of Radiotherapy, ULS Braga, 4710-243 Braga, Portugal; 7 Unidade de Cancro do Pulmão, CUF Oncologia, Hospital CUF Porto, 4100-180 Porto, Portugal; 8 Unidade de Tumores Cerebrais, CUF Oncologia, Hospital CUF Porto, 4100-180 Porto, Portugal; 9 Department of Medical Oncology, ULS Santo António, 4099-001 Porto, Portugal; 10 Oncology Research Unit (UMIB—Unit for Multidisciplinary Research in Biomedicine), ICBAS—School of Medicine and Biomedical Sciences, University of Porto, 4050-313 Porto, Portugal; 11 Instituto de Saúde Baseada na Evidência (ISBE), 1649-028 Lisbon, Portugal; 12 Department of Medical Affairs, J&J Innovative Medicine, 2740-298 Lisbon, Portugal; 13 Department of Pulmonology, ULS Coimbra, 3004-561 Coimbra, Portugal; 14 Department of Pulmonology, Instituto Português de Oncologia de Lisboa, 1099-023 Lisbon, Portugal; 15 Department of Medical Genetics, Faculdade de Medicina da Universidade de Lisboa, 1649-028 Lisbon, Portugal; 16 Department of Pulmonology, ULS São João, 4200-319 Porto, Portugal; 17 Faculty of Medicine, University of Porto, 4200-319 Porto, Portugal; 18 Institute of Molecular Pathology and Immunology of the University of Porto (IPATIMUP), 4200-135 Porto, Portugal

**Keywords:** brain, metastasis, non-small-cell lung cancer, NSCLC, EGFR-mutant NSCLC

## Abstract

Brain metastases represent a major clinical challenge in the management of non-small-cell lung cancer, particularly in patients with epidermal growth factor receptor mutations, constituting a leading cause of morbidity and mortality. In recent years, significant advancements have been made in both local therapies and systemic treatments. Nevertheless, managing central nervous system disease remains complex, encompassing challenges such as optimal therapy sequencing, resistance mechanisms, oligoprogression, the role of the blood–brain barrier, diagnostic strategies, and the influence of the brain tumor microenvironment. Despite advances, brain metastases in non-small-cell lung cancer remain an unmet need. Local treatments like radiotherapy are effective but carry risks of long-term neurological side effects, while the number and burden of brain metastases remain key prognostic factors. Surgical resection may be considered even in patients with multiple brain metastases to improve clinical status, enable further therapy, and enhance survival and quality of life. With the extension of survival afforded by systemic therapies, optimizing the balance between treatment efficacy and quality of life has become critical. Integrating patient perspectives is essential in navigating these complex clinical decisions.

## Simple Summary

Brain metastases are a common and clinically significant complication of EGFR-mutant non-small-cell lung cancer (NSCLC), substantially affecting prognosis, neurological function, and quality of life. This review examines the key challenges in their management, including the choice and integration of systemic therapies and radiotherapy, as well as the expanding role of CNS-active targeted agents and antibody-based treatments. It also explores the emerging utility of liquid biopsy, particularly cerebrospinal fluid analysis, for guiding treatment decisions. In addition, it addresses the biological determinants of CNS metastasis and treatment response, including CNS barriers and microenvironmental influences. Overall, the review highlights the importance of individualized, multidisciplinary treatment strategies that balance intracranial disease control, systemic disease management, neurotoxicity, and patient quality of life, while underscoring the need for prospective studies to define optimal therapeutic approaches in this rapidly evolving field.

## 1. Introduction

**Figure 1:**
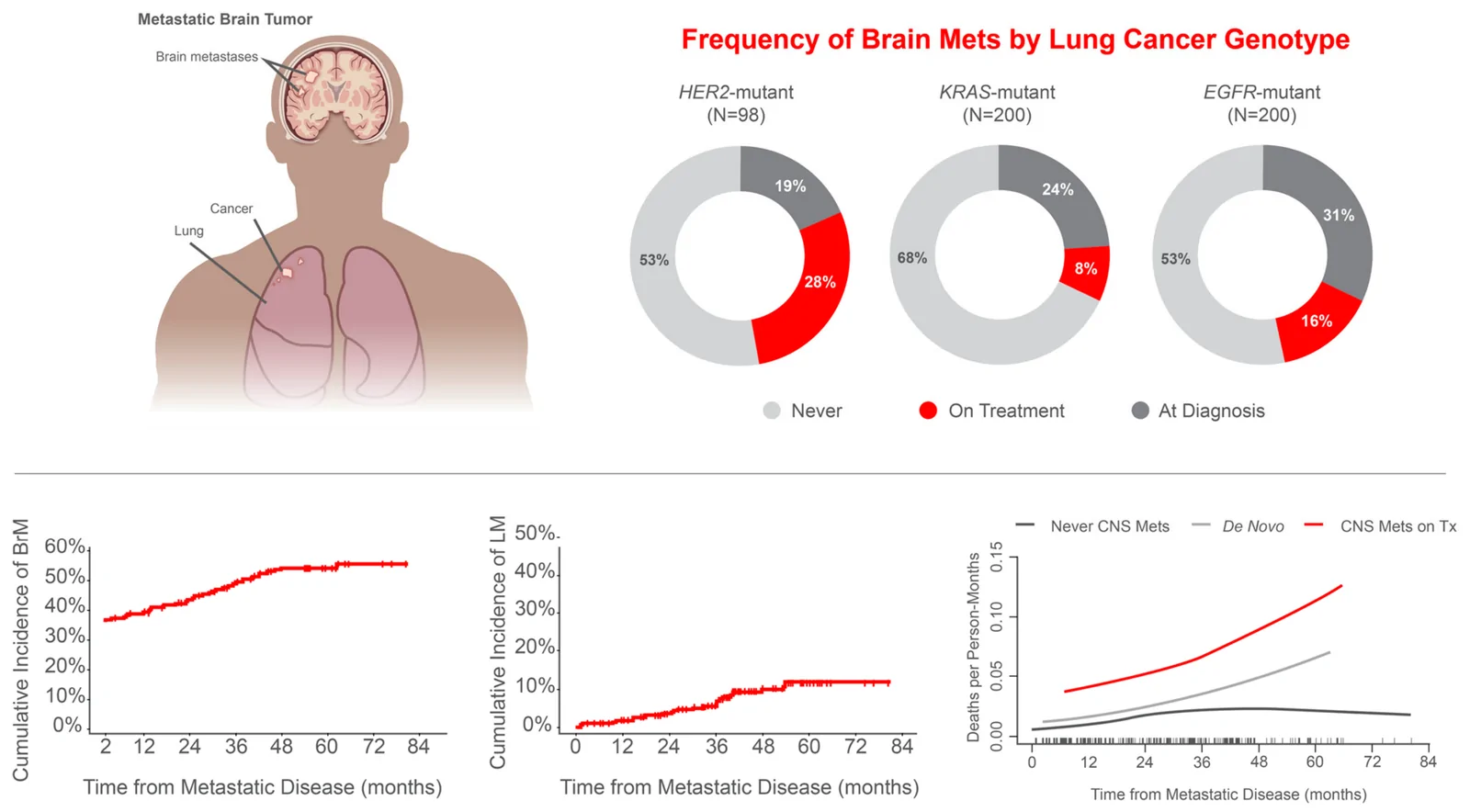
Frequency and incidence of brain metastases in lung cancer [1,2].

Brain metastases (BMs) pose a significant clinical challenge in the management of non-small-cell lung cancer (NSCLC), particularly in patients harboring epidermal growth factor receptor mutations (EGFRms), among whom the incidence can be notably high (Figure 1) [1,2]. The development of BM and leptomeningeal disease (LMD) often leads to a poor prognosis and significantly impairs patients’ quality of life, underscoring the critical need for optimized therapeutic strategies [2,3]. In recent years, there have been rapid advances in both local treatments, such as stereotactic radiosurgery (SRS), and systemic therapies, including newer-generation tyrosine kinase inhibitors (TKIs) with improved central nervous system (CNS) penetration [1,4,5,6,7] and emerging antibody-based treatments [8,9]. However, navigating the complexities of CNS disease management remains an area of active investigation and debate. Key open questions include the optimal sequencing of therapies, managing resistance and oligoprogression [10], understanding the role of the blood–brain barrier (BBB) [11], leveraging diagnostic tools such as liquid biopsy [11,12], and accounting for the unique brain tumor microenvironment (TME) [9,13].

To address these evolving challenges, a panel of experts identified key questions regarding the current management of BM in NSCLC. This review synthesizes recent evidence to examine these critical areas and provide insights into optimizing treatment strategies for this patient population.

## 2. Methods

To identify the most relevant controversies and key knowledge gaps in the management of BM and LMD in patients with EGFRm NSCLC, three face-to-face meetings were held in 2024. Each meeting convened a dedicated panel of key opinion leaders from one of three specialties (Medical Oncology, Pneumology, or Radiology/Radiation Oncology) to reflect the multidisciplinary nature of BM management. Panel members were selected for their recognized clinical and research expertise in the topic and to represent diverse medical specialties and geographic regions. Before each meeting, participants received a curated selection of relevant research to facilitate evidence-informed discussions.

Each three-hour meeting followed a semi-structured discussion guide designed to facilitate the exchange of expert perspectives and to identify areas of clinical uncertainty. Through iterative discussion, the panel reached agreement on six priority questions that represent the most relevant unresolved challenges in the field:

1. Should upfront systemic or local therapy be preferred for BM in EGFRm NSCLC?
2. Can novel systemic therapies replace radiotherapy for untreated BM in EGFRm NSCLC?
3. Can liquid biopsy guide the management of BM and LMD in EGFRm NSCLC?
4. How can CNS penetration of systemic therapies be improved?
5. What is the role of the TME in BM and LMD?
6. What biological factors drive organ-specific metastasis?

After identifying these priority questions, a targeted, non-systematic narrative literature review was conducted in PubMed/MEDLINE using broad search terms, including “NSCLC” and “metastasis,” combined with question-specific keywords tailored to each topic. The final literature search was conducted in February 2025. To provide a contemporary perspective, English-language publications from January 2020 to February 2025 were considered, irrespective of study design. References were selected for relevance to each predefined clinical question, with priority given to high-quality evidence, including clinical practice guidelines, systematic reviews, meta-analyses, randomized clinical trials, and pivotal prospective studies. Additional landmark publications deemed essential were included when appropriate. The retrieved evidence was narratively synthesized to contextualize current knowledge gaps and identify priorities for future research. Given the narrative nature of this review, no formal assessment of evidence quality was performed. The strength of the available evidence is described qualitatively throughout the narrative, distinguishing findings supported by clinical trials from those based on real-world studies, translational research, or expert opinion, where appropriate and applicable.

## 3. Results

### 3.1. Should Upfront Systemic or Local Therapy Be Preferred for BM in EGFRm NSCLC?

EGFRm NSCLC has a well-recognized proclivity for CNS involvement, and the introduction of highly CNS-penetrant systemic therapies has substantially reshaped treatment paradigms for BM. Third-generation EGFR TKIs, most notably osimertinib, demonstrate superior blood–brain barrier penetration, achieve higher intracranial response rates, and confer prolonged intracranial progression-free survival (PFS) and improved overall survival (OS), compared with earlier-generation agents [1,4,14,15,16,17]. For asymptomatic patients with small or multiple BMs, frontline systemic therapy with a CNS-active TKI is widely endorsed because it addresses both intracranial and extracranial disease and frequently permits safe deferral of cranial irradiation [14]. When intracranial progression occurs on osimertinib 80 mg, contemporary expert consensus advocates continuing osimertinib while adding focal SRS rather than initiating whole-brain radiotherapy (WBRT) immediately. Dose escalation to 160 mg can be considered in selected situations and is often preferred for LMD when supported by clinical judgment and availability [18]. Combination systemic approaches have further expanded options for CNS disease control. The FLAURA2 trial demonstrated that adding chemotherapy to osimertinib significantly reduced CNS progression and markedly increased CNS complete remission rates among patients with measurable lesions ≥ 1 cm (48% vs. 16%) [19]. Similarly, MARIPOSA reported that first-line amivantamab plus lazertinib prolonged intracranial durability and halved the 3-year intracranial progression risk relative to osimertinib, despite a comparable intracranial objective response rate (ORR) [20]. These data highlight the emerging role of antibody-containing CNS-active regimens and support prioritizing CNS-penetrant systemic molecules when clinically appropriate.

Local therapies remain pivotal when immediate decompression or high-precision local control is required. SRS is the modality of choice for limited intracranial disease and for symptomatic or large lesions with mass effect, as it provides high rates of local control while sparing uninvolved brain tissue [14,21]. Neurosurgical resection remains indicated for accessible lesions that cause mass effect or require histologic confirmation. By contrast, WBRT, although effective for widespread intracranial involvement, has an increasingly limited role, because of its well-documented and often irreversible neurocognitive sequelae. Accordingly, WBRT is generally reserved for patients with diffuse CNS disease that is not amenable to focal techniques [15,21,22]. Real-world data showed that upfront EGFR-TKI with deferred radiotherapy was associated with inferior overall survival. In contrast, stereotactic radiosurgery followed by EGFR-TKI yielded the longest OS while avoiding the risk of neurocognitive decline associated with whole-brain radiotherapy [23]. However, analyses that include modern CNS-penetrant agents yield heterogeneous results. Some reports indicate no clear OS advantage to adding upfront SRS to CNS-penetrant TKIs [23]. Other studies, including the TURBO trial, demonstrate improved time to CNS progression and superior local control with upfront SRS, particularly for lesions ≥ 1 cm [23,24,25]. These findings collectively suggest that lesion size (notably ≥1 cm), symptom burden, and expected survival are critical determinants of whether upfront focal therapy provides an incremental survival or quality-of-life benefit over systemic monotherapy.

CNS oligoprogression, defined as limited intracranial progression in the context of otherwise controlled systemic disease, has become a common clinical pattern as systemic therapies achieve durable extracranial control. Although the optimal management strategy remains uncertain, evidence from other oncogene-driven malignancies, such as human epidermal growth factor receptor 2 (HER2)-positive breast cancer, supports combining focal local therapy with continuation of the current effective systemic treatment. This approach aims to preserve extracranial disease control and delay wholesale changes in systemic therapy [10,26]. In this context, focal treatments may include SRS to the progressing sites, or, for selected lesions, neurosurgical resection followed by SRS to the resection cavity. When a change in systemic therapy is eventually required because of broader resistance, completing focal radiation before initiating the next agent is often prudent to minimize overlapping toxicities and optimize local control [26,27]. The biological rationale for such selective local interventions is supported by evidence that BM frequently exhibits distinct genomic profiles compared with those of primary or extracranial sites. These profiles include higher tumor mutational burden, a greater fraction of the genome altered, more frequent whole-genome duplication, and enrichment of cell cycle pathway alterations [28,29]. These genomic and microenvironmental distinctions suggest that intracranial lesions may undergo independent clonal evolution, potentially explaining why site-specific focal interventions remain necessary for a subset of patients even when systemic disease remains otherwise wellcontrolled.

Radiation-related neurotoxicity is a major trade-off that must be weighed carefully, especially as effective systemic therapies lengthen survival. WBRT is associated with measurable declines in neurocognitive function and reductions in quality of life, effects that are of consequence for patients with longer life expectancy [14,15]. Although SRS is less damaging to global cognitive function, it is not without risk: focal radiation necrosis, symptomatic edema, and cumulative toxicity from multiple or repeated treatments can cause substantial morbidity [15,22,30]. Given the enhanced CNS activity of contemporary systemic agents, there is growing justification for deferring or avoiding cranial irradiation when clinically appropriate to minimize late neurotoxicity. When WBRT cannot be avoided, hippocampal sparing techniques and neuroprotective pharmacotherapy such as memantine should be employed to mitigate cognitive decline [15].

Despite the advances summarized above, several critical evidence gaps remain. Prospective, randomized trials are needed to identify which patients derive an OS benefit from upfront focal therapy in the context of potent CNS-penetrant systemic regimens, and to determine whether antibody plus TKI combinations can reliably obviate the need for radiation in selected cohorts without compromising long-term outcomes. Biomarker-driven studies that explicitly address the distinct genomics of BM are needed to refine patient selection for local interventions and to elucidate mechanisms of intracranial resistance. Mechanistic preclinical work should also clarify whether combined TKI–radiation regimens produce additive or synergistic antitumor effects in EGFRm disease [15]. In clinical practice, treatment selection should be individualized within a multidisciplinary tumor board, taking into account lesion size and number, symptom severity, performance status, anticipated survival, resistance biology, and patient preferences.

In summary, for most patients with EGFRm NSCLC and asymptomatic or small, multiple BMs, initial management should prioritize systemic therapy with CNS-penetrant agents (third-generation TKIs and emerging CNS-active antibody combinations). These agents target both intracranial and extracranial disease while minimizing immediate exposure to radiation and its associated neurocognitive risks [1,4,14,15,16,17,18,19,20]. Upfront local therapy, consisting of surgical resection or, for unresectable lesions, fractionated stereotactic radiotherapy, remains generally recommended for patients with symptomatic, large, or life-threatening brain metastases. While SRS is effective for smaller targets, larger volumes often require fractionation to mitigate the risk of radionecrosis and to reduce exacerbation of perilesional edema. Local consolidation should also be strongly considered for selected patients with limited intracranial disease (particularly lesions ≥ 1 cm), as durable local control is likely to yield clinical benefit [21,22,23,24,25]. WBRT should be reserved for diffuse, unresectable intracranial disease and, when used, combined with neuroprotective strategies [15]. Ultimately, multidisciplinary, biomarker-driven decision-making and prospective clinical trials are urgently needed to optimize selection between upfront SRS and systemic-first strategies in the era of highly CNS-penetrant therapies [1,4,14,15,16,17,18,19,20,21,22,23,24,25,26,27,28,29,30,31,32,33].

### 3.2. Can Novel Systemic Therapies Replace Radiotherapy for Untreated BM in EGFRm NSCLC?

Patients with EGFRm NSCLC have a notably high incidence of BM, up to 70%, which significantly exceeds that in EGFR wild-type NSCLC. Current treatment options include EGFR TKIs, WBRT, SRS, and chemotherapy; the optimal management and sequencing remain undefined [5]. Newer-generation EGFR TKIs, such as osimertinib, have demonstrated improved BBB penetration and significant intracranial activity, which may contribute to improved OS [5]. However, BM remains a major clinical challenge, substantially contributing to disease burden and poor outcomes.

Recently, antibody-based regimens, including antibody–drug conjugates, bispecific T-cell engagers, and bispecific antibodies, have emerged as promising options for managing EGFRm NSCLC with BM [8].

Human epidermal growth factor receptor 3 (HER3) is frequently overexpressed in BM of NSCLC (73%) and breast cancer (75%), often exceeding expression levels at extracranial sites [34,35]. This makes it a critical target, particularly because HER3 has been shown to facilitate CNS colonization by cancer cells. Although data on the CNS efficacy of antibody–drug conjugates remain limited (a critical consideration in oncogene-driven NSCLC), recent clinical trials have shown promising results.

In the HERTHENA-Lung01 trial, patritumab deruxtecan demonstrated an intracranial ORR of 33% among 30 patients with untreated baseline BM [36]. The median OS in this trial for patients with advanced EGFRm NSCLC without LMD was 11.9 months, while a separate cohort of patients with LMD had median OS of 10.5 months [37]. Further investigation is underway in the TUXEDO-3 trial (NCT05865990) and the PARAMETer trial (NCT05620914). However, patritumab deruxtecan is not approved for the treatment of NSCLC (HERTHENA-Lung02-NCT05338970 did not meet its key secondary endpoint), which limits its use in the clinical setting.

Similarly, biomarker-agnostic strategies targeting trophoblast cell surface antigen 2 (TROP2) are being explored after osimertinib failure [38,39,40]. In the TROPION-Lung05 study, datopotamab deruxtecan (Dato-DXd) demonstrated comparable systemic efficacy regardless of baseline BM status. Among patients with measurable intracranial lesions, the ORR was 22% (18% in EGFRm) with a disease control rate of 72% [41]. Preclinical models suggest that Dato-DXd can penetrate the BBB to mediate these effects [42], warranting further study in trials such as TUXEDO-2 (NCT05866432) and DATO-BASE (NCT06176261). Dato-DXd received FDA approval for previously treated EGFR-mutant NSCLC based on TROPION-Lung05 and TROPION-Lung01.

The bispecific EGFR/MET antibody amivantamab has shown robust intracranial efficacy. In the MARIPOSA-2 trial, the combination of amivantamab and chemotherapy significantly delayed CNS recurrence. Among patients with BM who had no prior radiotherapy, the median intracranial PFS was 11.1 months with amivantamab-lazertinib-chemotherapy versus 6.3 months with chemotherapy alone (hazard ratio 0.36) [21].

Amivantamab plus lazertinib also shows antitumor activity in patients who develop new or progressive brain or leptomeningeal metastases. Patients with BM had an ORR of 50%, median PFS of 5.8 months, and median OS of 17.4 months. Those with LMD had an ORR of 33%, median PFS of 7.8 months, and median OS of 14.4 months [43]. Despite being a large molecule, amivantamab demonstrates clinical efficacy, potentially mediated by direct antitumor effects and/or immune-mediated mechanisms, challenging the traditional view that large antibodies generally lack BBB penetration [21]. Worldwide regulatory approvals support amivantamab-based regimens in EGFR-mutant NSCLC.

The efficacy of ICIs in NSCLC patients with brain metastases who lack actionable alterations provides proof of concept that therapeutic antibodies can achieve meaningful intracranial activity, supporting the development of other antibody-based approaches in this setting [9]. By blocking inhibitory pathways, ICIs such as pembrolizumab and nivolumab enhance the immune response. In patients with NSCLC and BM, ICIs have demonstrated significant efficacy, with studies reporting disease control rates of 39% among those with asymptomatic BM [44].

**Figure 2:**
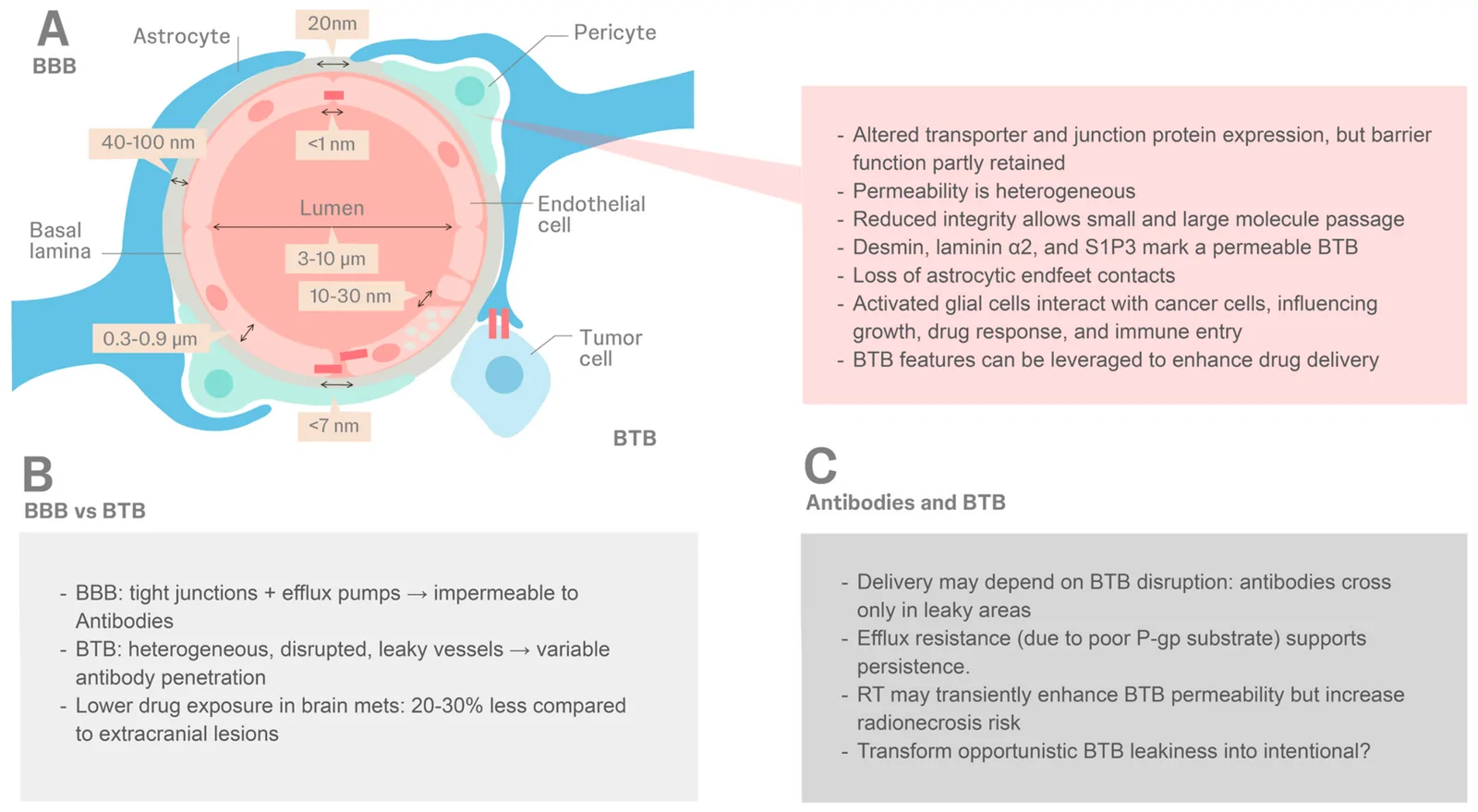
The blood–brain barrier (BBB) versus blood-tumor barrier (BTB) and implications for drug delivery. (**A**) A schematic comparison of the BBB and BTB, (**B**) key functional differences, and (**C**) therapeutic implications. Although BBB and BTB disruption may enhance drug penetration, heterogeneous permeability across metastatic lesions may not ensure adequate intracranial drug exposure.

The efficacy of large molecules challenges the historical dogma that the BBB is impermeable to antibodies. Evidence suggests that in patients with BM, the BBB is inherently compromised, forming a “blood–tumor barrier” (BTB) (Figure 2). This heterogeneous vasculature is marked by disrupted neurovascular units, altered transporter expression, and loss of astrocytic end-foot connections [12]. Importantly, although BBB disruption may facilitate drug delivery, permeability is highly heterogeneous across brain metastases and does not necessarily provide sufficient intracranial drug exposure or uniform drug distribution within metastatic lesions.

This structural compromise may facilitate the passage of therapeutic antibodies. Furthermore, combination therapies pairing ICIs with anti-angiogenic agents such as bevacizumab may further enhance drug delivery by normalizing tumor vasculature [45]. Bevacizumab, by inhibiting vascular endothelial growth factor (VEGF), suppresses angiogenesis and has shown promise in preclinical studies by suppressing the growth of established BM [46].

From a clinical perspective, this combination is particularly valuable when strategies to reduce peritumoral vasogenic edema are needed. Because corticosteroids can reduce the efficacy of ICIs, the bevacizumab plus ICI regimen is a crucial alternative for managing edema without compromising the immune response.

Before broadly considering these new molecules as alternatives to radiotherapy, caution is warranted. Robust data on the CNS efficacy of antibody–drug conjugates remain limited [46], and the optimal integration of these therapies with TKIs, chemotherapy, or radiotherapy remains an open question [22]. Multiple novel agents and combinations are under investigation, including in phase III studies, such as zipalertinib, sunvozertinib, furmonertinib, and combinations with osimertinib plus savolitinib, which also show encouraging brain activity [47]. Several ADC-based regimens, including izalontamab brengitecan, sacituzumab tirumotecan, and telisotuzumab adizutecan, continue to be evaluated, with additional clinical data expected in this setting.

However, the shift toward antibody-based regimens offers a potential avenue to reduce cumulative neurological toxicity associated with repeated radiotherapy while improving intracranial control [5,20,22,48]. As research evolves, clarifying the role of these agents will be essential to optimizing treatment strategies.

### 3.3. Can Liquid Biopsy Guide the Management of BM and LMD in EGFRm NSCLC?

Liquid biopsy refers to a set of minimally invasive techniques used to analyze biomarkers, such as cell-free DNA, extracellular vesicles, microRNAs, circulating tumor cells, and tumor-derived metabolites, detected in bodily fluids such as blood or cerebrospinal fluid (CSF) [49]. While activation of the EGFR pathway drives oncogenesis in 15% to 40% of NSCLC cases [23], brain metastatic sites are highly divergent from primary tumors despite sharing a common ancestor [50].

**Figure 3:**
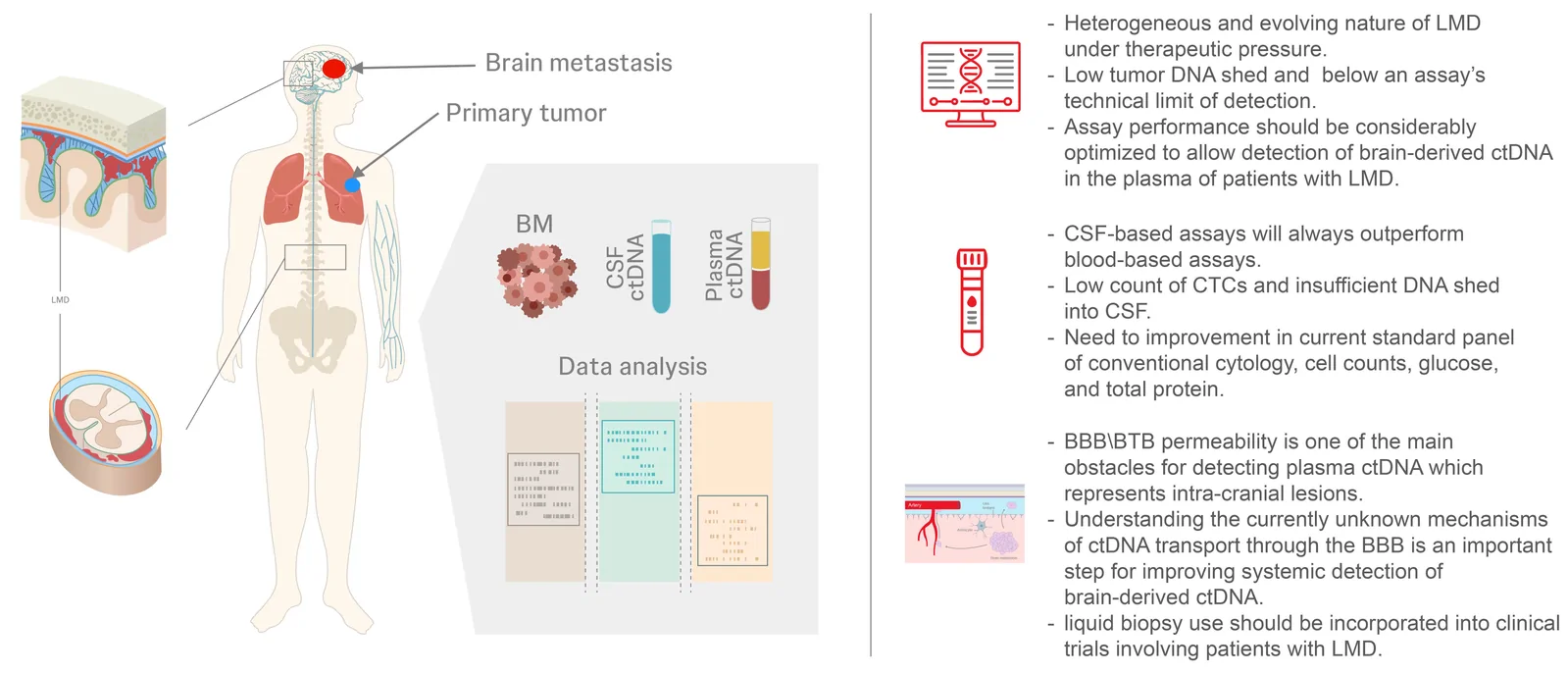
Challenges for liquid biopsy development in patients with CNS metastases from NSCLC.

In the context of systemic disease, circulating tumor DNA (ctDNA) levels in plasma are strongly prognostic for disease-free and distant metastasis-free survival, and serve as indicators of micrometastases. However, no reliable inferences can be drawn regarding CNS disease due to the BBB. Data suggest that “ctDNA shedding” (detecting the driver EGFR mutation in plasma) is associated with a higher incidence of BM. Conversely, patients who are “non-shedders” before and after osimertinib treatment and who have no BM show longer post-progression survival [51,52]. Nevertheless, for patients with stable or active BM, data on using plasma liquid biopsy to guide therapy remain limited (Figure 3).

A particularly challenging scenario arises in patients with metastases confined to the brain or LMD, where plasma liquid biopsy often fails due to the BBB. In these cases, CSF is emerging as a valuable alternative [53]. Its proximity to brain tumors and low baseline cellularity reduces background noise, enhancing sensitivity for detecting tumor-derived material compared with plasma [52].

Studies indicate that CSF ctDNA provides a more comprehensive driver gene profile during CNS progression. For instance, EGFR mutations were detected in 85.5% of LMD cases in CSF, including T790M in 16.1% [54]. Comparative studies show EGFR detection rates of 82% in CSF versus 45% in matched plasma, with similar superiority observed for anaplastic lymphoma kinase (ALK) and ROS proto-oncogene 1, receptor tyrosine kinase (ROS1) [55]. Furthermore, mutant-allele frequencies are significantly higher in CSF [55], and some studies report a 100% detection rate for driver mutations in CSF samples [56].

Consequently, the National Comprehensive Cancer Network (NCCN) guidelines state that, when available, CSF liquid biopsy may be considered an adjunct to conventional CSF cytology for patients with leptomeningeal disease [57].

The role of CSF in LMD is multifaceted. CSF cytology remains the established diagnostic approach for evaluating suspected LMD [58]. However, sensitivity can be low, often requiring multiple samples (up to three) to improve diagnostic yield [59,60,61]. In approximately 25% of cases, positive cytology does not align with clinical features or with biochemical markers such as glucose and protein [62].

When cytology is negative but suspicion remains high, CSF ctDNA analysis becomes critical. It can reveal actionable mutations missed by blood samples and provide evidence of malignancy when magnetic resonance imaging is inconclusive [52,63,64]. Conversely, plasma liquid biopsies have not achieved sufficient diagnostic accuracy for LMD and are not currently recommended [61,65,66,67].

CSF analysis is crucial for tailoring targeted therapies. Genotyping can identify resistance mechanisms, such as T790M or specific EGFR 19 deletions, informing treatment strategies more accurately than plasma [64]. For example, the BLOSSOM trial demonstrated the intracranial efficacy of osimertinib 80 mg in LMD, with pharmacokinetic analysis showing a CSF-to-free-plasma ratio of 22% [68]. Similarly, the combination of amivantamab and lazertinib has shown promise, with 64% of LMD patients showing a decrease in CSF circulating tumor cells [69].

Although head-to-head comparisons are limited, single-cell sequencing of circulating tumor cells may provide better characterization of mutational diversity than cell-free DNA [65,70,71]. However, interpreting CSF findings requires caution. Extracranial resistance mechanisms cannot be assumed to exist in the CNS without direct evidence [72]. Furthermore, CNS progression on TKIs may result from low drug exposure rather than true resistance, potentially supporting continuation of TKIs with high BBB penetration alongside systemic treatments [73].

Despite its promise, CSF analysis faces challenges. Technical factors, including sampling site, volume, and processing, affect accuracy [61]. Interpreting CSF cell-free DNA is complex; molecular variations may reflect clonal randomness rather than dominant drivers, and “lessons from plasma” warn that high variant allele frequencies could indicate non-tumor sources [72]. Additionally, longitudinal CSF assessment is invasive and often not feasible. Response assessment in LMD remains challenging because of the lack of validated imaging criteria and the difficulty in distinguishing cancer progression from neurotoxicity or comorbidities [65].

From a practical standpoint, the clinical roles of plasma and CSF liquid biopsies differ substantially and should be considered complementary. From a clinical perspective, plasma and CSF liquid biopsies provide complementary information in patients with EGFRm NSCLC and CNS involvement. Plasma ctDNA remains useful for systemic disease monitoring and for identifying resistance mechanisms outside the CNS. Given its minimally invasive nature, ease of collection, and suitability for serial assessment, plasma ctDNA remains the preferred liquid biopsy approach in routine clinical practice. However, its ability to detect isolated CNS progression is limited by the BBB. In contrast, CSF ctDNA provides a more accurate representation of the molecular landscape of brain metastases and leptomeningeal disease, thereby improving detection of actionable alterations and resistance mechanisms when CNS progression occurs. Nevertheless, CSF sampling requires lumbar puncture, making it more invasive and less practical for routine longitudinal monitoring. Accordingly, plasma ctDNA is best suited for systemic disease surveillance. In contrast, CSF ctDNA may be particularly valuable for diagnosing CNS progression, characterizing the molecular landscape of BM and LMD, and identifying CNS-specific resistance mechanisms.

Overall, liquid biopsy, particularly ctDNA analysis, offers a minimally invasive approach to monitoring disease progression and guiding treatment for patients with EGFRm NSCLC and CNS involvement [74]. While challenges in standardization and sensitivity remain, integrating CSF analysis into clinical practice is a significant step toward optimizing the management of this complex patient population.

### 3.4. How Can CNS Penetration of Systemic Therapies Be Improved?

The development of CNS therapeutics is challenging because of the BBB, a complex interface that maintains CNS homeostasis by regulating the passage of nutrients (e.g., amino acids, glucose) while blocking harmful molecules such as neurotoxins [15,27]. However, the BBB also restricts the entry of certain drugs and large biopharmaceuticals into the brain [11]. For instance, after parenteral administration of therapeutic antibodies, their concentration in the brain is only 0.01–0.10% of plasma levels. These low antibody levels in the brain may still produce pharmacological effects in certain cases, depending on the molecular target and the antibody’s interaction dynamics. In many instances, however, achieving a higher brain concentration of the antibody would be advantageous, reducing the required dose and enhancing the therapeutic index [11].

BMs are a hallmark of EGFRm (Figure 1) and ALK fusion in NSCLC, with a higher propensity for brain involvement than in EGFR wild-type cases [75]. Their presence negatively impacts clinical outcomes, as patients treated with first- or second-generation TKIs often experience shorter PFS [3]. Improving CNS penetration of systemic therapies is essential for effectively managing BM and LMD, especially in patients with EGFRm. For treating metastatic brain disease, BBB permeability is considered desirable to increase clinical efficacy. The development of brain-penetrant molecules for oncogene-defined NSCLC is an active area of research [6,27,30].

The emergence of novel TKIs with enhanced CNS penetration has led to a paradigm shift in treating NSCLC patients with BM. These newer-generation TKIs have demonstrated promising intracranial activity in preclinical and clinical studies [6]. For instance, ALK inhibitors such as alectinib, lorlatinib, and brigatinib, as well as the third-generation EGFR inhibitor osimertinib, lazertinib, and aumolertinib, have shown improved CNS penetration and efficacy compared with earlier generations [7]. Some of these agents have achieved CNS ORRs of 70–80% in patients with targetable molecular alterations [30].

Notably, high-dose pulsatile therapy has been explored to improve CNS penetration. In a case study, high-dose pulsatile crizotinib (1000 mg/day) administered on an alternating-day schedule (one day on, one day off) led to a significant CNS response and prolonged time to neurological progression in a patient with NSCLC and multiple intracranial metastases. According to the European Society for Medical Oncology (ESMO) expert consensus recommendations, the standard approach for patients with intracranial progression despite osimertinib 80 mg is to continue the current dose of osimertinib. An increased dose of osimertinib 160 mg may be considered if accessible [76]. This approach may help overcome pharmacokinetic and biodynamic resistance associated with CNS metastases.

The limited brain penetration of certain clinically used EGFR TKIs may be attributed to their physicochemical properties, which fall outside the optimal ranges for BBB penetration, including molecular weight, hydrogen bond donors and acceptors, polar surface area, and rotatable bonds. These properties also affect interactions with P-glycoprotein (P-gp) and the breast cancer resistance protein, both critical efflux transporters at the BBB, further restricting drug exposure in the CNS [77].

Intrathecal administration is another approach. In a retrospective cohort study of 23 patients with NSCLC and LMD treated with the novel EGFR-TKI furmonertinib, administered intrathecally alongside methotrexate or pemetrexed, the median PFS was 10.8 months, while the median OS was not reached [78]. Although not specifically targeting NSCLC patients, several strategies have emerged to enhance drug delivery to the CNS, including targeted therapies, innovative drug delivery systems, and techniques to temporarily disrupt the BBB. One promising approach uses targeted therapies designed to cross the BBB. For instance, early research on next-generation EGFR-TKIs such as zorifertinib, approved in China since 2024, has shown improved CNS penetration compared to first-generation TKIs like gefitinib and erlotinib [79,80]. These newer-generation agents might be particularly beneficial for patients with EGFRm NSCLC who develop BM, as they could effectively target and inhibit tumor growth within the CNS [81,82]. Zorifertinib exhibits superior BBB penetration compared with earlier-generation EGFR-TKIs, enhancing its potential efficacy in treating BM in patients with EGFRm NSCLC. This enhanced CNS distribution is primarily due to its low affinity for efflux transporters such as P-gp and breast cancer resistance protein. These transporters are highly expressed at the BBB and actively restrict the entry of many therapeutic agents into the CNS. Unlike first- and second-generation EGFR-TKIs, which these transporters efficiently exclude from the brain, zorifertinib avoids this efflux mechanism and achieves sustained intracranial exposure. Furthermore, its physicochemical properties—moderate molecular weight, balanced lipophilicity, and favorable ionization state—promote passive diffusion across the BBB. Preclinical studies have demonstrated a high unbound brain-to-plasma ratio (Kp, uu), indicating effective free drug availability in the CNS. These findings, together with emerging clinical data showing intracranial responses, position zorifertinib as a promising treatment for EGFRm NSCLC with BM [80,83].

Beyond targeted therapies, emerging investigational strategies include nanoparticle- and liposome-based delivery systems designed to enhance drug delivery across the BBB. Nanoparticles can be engineered to improve BBB penetration by modifying their surface properties or by using specific ligands that facilitate transport across the barrier [84]. For example, incorporating targeting ligands such as arginine-glycine-aspartic acid peptides into nanoparticles has been shown to enhance their penetration into brain tissues [85]. Similarly, liposomal formulations can protect encapsulated drugs from degradation and improve their bioavailability in the CNS [86].

Another investigational approach is convection-enhanced delivery, which enables direct infusion of therapeutic agents into the brain’s interstitial space, bypassing the BBB. Convection-enhanced delivery uses a pressure gradient to distribute drugs uniformly throughout the target area, achieving higher local concentrations than conventional systemic administration [87,88]. This method has been explored in experimental settings for the treatment of glioblastomas and could be adapted for use in NSCLC with BM [87,88]. Recent advancements in catheter design, such as step-design catheters, have further improved the efficacy of convection-enhanced delivery by minimizing reflux and enhancing drug distribution [87].

Moreover, transient BBB disruption is a promising investigational strategy to enhance drug delivery to the CNS. Methods such as focused ultrasound combined with microbubble technology have been explored to transiently open the BBB, thereby increasing the penetration of therapeutic agents into the CNS [89]. This approach has shown promise in preclinical models and may be applicable in clinical settings for patients with BM. Another therapeutic approach is Tumor Treating Fields, a non-invasive cancer therapy that delivers low-intensity, intermediate-frequency alternating electric fields directly to the tumor. It has been demonstrated to disrupt mitotic spindle assembly by interfering with the significant dipole moment of microtubules, leading to metaphase arrest, prolonged mitosis, abnormal daughter cell formation, and ultimately cell death [90]. Electromagnetic fields are used to disrupt mitotic spindles during anaphase, thereby inducing apoptosis in tumor cells [91]. Prospective clinical trials are warranted to validate this approach and to define its potential role in managing CNS metastases.

Improving CNS penetration of systemic therapies in NSCLC involves a multifaceted approach that includes optimizing drug properties, modulating the BBB, leveraging nanotechnology, and integrating combination therapies. Although these approaches remain largely investigational, ongoing research into emerging technologies and therapeutic strategies holds promise for improving the management of CNS involvement in NSCLC and other cancers prone to CNS metastasis.

### 3.5. What Is the Role of the TME in BM or LMD?

The brain TME plays a significant role in both promoting and inhibiting BM and LMD in NSCLC patients. The brain TME is unique because of its composition and interactions among various cell types, which can either support or impede the growth and spread of metastatic cells. Several factors within the brain TME, including immune cells, astrocytes, microglia, the extracellular matrix (ECM), and the BBB, are involved in these processes [13].

As noted above, there are promoting factors within the TME. Expression of specific chemokine receptors, such as C-X-C chemokine receptor type 4 (CXCR4), has been associated with brain metastasis in NSCLC. Studies have shown that high levels of CXCR4 expression correlate with an increased likelihood of BM [92,93]. This suggests that the C-X-C motif chemokine ligand 12 (CXCL12)/CXCR4 axis facilitates tumor cell migration and invasion into the brain. This interaction not only promotes the establishment of metastases but also shapes the immune landscape within the brain, potentially creating a more favorable environment for tumor growth. The presence and polarization of immune cells within the TME can also significantly influence tumor behavior. For instance, infiltration of tumor-associated macrophages has been shown to promote BM by enhancing angiogenesis and supplying growth factors that support tumor survival [94]. In contrast, a suppressed immune microenvironment characterized by a predominance of M2 macrophages can facilitate tumor progression and metastasis [95]. The balance among immune cell types, including T cells and macrophages, can therefore determine the metastatic potential of NSCLC cells in the brain. The TME is also shaped by the composition and remodeling of the ECM. Cancer-associated fibroblasts play a significant role in this process, contributing to ECM changes that can enhance tumor cell invasion and survival [96,97,98]. ECM remodeling can create a permissive environment for metastasis by facilitating tumor cell migration and providing biochemical signals that promote growth.

Among inhibitory factors in the TME, the BBB serves as a protective barrier that can inhibit the entry of therapeutic agents and metastatic cells into the brain. While some tumor cells can exploit pathways to breach the BBB, the integrity of this barrier can act as a significant obstacle to metastasis. The ability of NSCLC cells to penetrate the BBB is influenced by their molecular characteristics and the presence of specific signaling pathways that facilitate this process [13]. The immune system plays a dual role within the TME. While some immune cells can promote tumor growth, others can exert antitumor effects. The presence of activated T cells and natural killer cells can inhibit the establishment and growth of metastases [95]. However, immune surveillance can be compromised by the immunosuppressive environment often found in BM, where factors such as programmed death-ligand 1 (PD-L1) expression can inhibit T-cell activity [99]. The TME can also influence the epigenetic landscape of tumor cells, thereby influencing their metastatic potential. For example, microRNAs such as miR-596-3p have been shown to suppress BM by modulating pathways that drive tumor growth and invasion [100]. This suggests that interactions within the TME can lead to changes in gene expression that either promote or suppress metastasis.

In summary, the TME in the brain plays a complex role in the development of BM and LMD in NSCLC patients. Factors such as chemokine signaling, immune cell dynamics, and ECM remodeling can promote metastasis, whereas the integrity of the BBB and immune surveillance can inhibit it. Understanding these mechanisms is crucial for developing targeted therapies to effectively manage BM in NSCLC.

### 3.6. What Biological Factors Drive Organ-Specific Metastasis?

The evidence supporting organotropism in NSCLC derives from multiple complementary lines of investigation, including clinical observations, genomic analyses, translational studies, and mechanistic preclinical models. This heterogeneity in evidence should be considered when interpreting the strength and disease-specific relevance of each proposed mechanism. Tumors that arise in different organs often show a preference for metastasizing to certain organs, a phenomenon known as ‘organotropism.’ This process reflects the interplay between circulatory and anatomical constraints, the metastatic microenvironment, and intrinsic tumor cell programs that enable survival beyond the primary site [101]. In NSCLC, particular attention has focused on mechanisms that may support CNS dissemination, including brain metastases and leptomeningeal disease. Clinical studies have further shown that oncogenic drivers may also contribute to organ-specific metastatic behavior. In NSCLC, actionable genomic alterations such as EGFR mutations and ALK rearrangements are associated with a higher incidence of brain metastases, supporting the concept that specific molecular subtypes have an inherent propensity for CNS dissemination [102]. Successful colonization of distant organs also requires tumor cell plasticity, self-sufficiency, and the ability to interact with and adapt to the local microenvironment by producing growth factors and by activating context-specific survival programs [102]. Translational studies using patient-derived CNS samples have begun to define molecular programs associated with leptomeningeal and brain metastatic disease. In an exploratory single-cell transcriptomic analysis of cerebrospinal fluid-derived tumor cells from patients with leptomeningeal metastases from lung adenocarcinoma, Ruan et al. analyzed 792 single-cell transcriptomes from five patients and three controls and identified a metastatic gene signature enriched for pathways involved in cellular metabolism, cell adhesion, and adaptation to the CNS microenvironment. Transcriptional heterogeneity was greater between patients than among individual cells from the same patient, highlighting substantial interpatient diversity. Although the small cohort size limits generalizability, these findings support the concept that CNS dissemination is accompanied by distinct adaptive transcriptional programs [103]. Similarly, using patient-derived xenograft models generated from matched primary lung adenocarcinomas and brain metastases, Zhang et al. identified several differentially expressed genes linked to metastatic progression. Among these, CKAP4, SERPINA1, SDC2, and GNG11 emerged as genes of interest associated with metastatic progression [104], warranting validation in larger clinical cohorts before any prognostic or therapeutic relevance can be inferred. These alterations suggest that tumor cells must acquire biological traits that support survival and adaptation within the target organ, including interactions with the local microenvironment, activation of growth-promoting pathways, and molecular programs enabling invasion and metastatic colonization. Epithelial–mesenchymal transition (EMT) is an evolutionarily conserved process essential for embryonic development and increasingly recognized as a contributor to cancer progression and metastasis. EMT-associated plasticity may facilitate several steps of the metastatic cascade, including loss of epithelial adhesion, acquisition of invasive properties, extracellular matrix remodeling, and adaptation to the brain microenvironment [105]. E-cadherin, which mediates cell adhesion, is notably diminished in patients with NSCLC who have BM, compared with those without, providing evidence for the link between EMT and BM development [106]. In NSCLC, vimentin overexpression, a canonical marker of EMT, has been associated with tumor dedifferentiation, invasive behavior, and increased metastatic potential [107]. Loss of epithelial features during metastatic development also leads to tumor stromal degradation due to the upregulation of matrix metalloproteases and plasminogen activators, promoting an invasive phenotype [108]. Elevated MMP-9 levels have been observed in lung cancer brain metastases, and experimental data suggest that MMP-9 may contribute to BBB transmigration and CNS colonization [109]. Despite significant advances, the molecular determinants of organ-specific metastasis remain incompletely characterized. In a distinct mechanistic experimental context, RBMX2 was recently shown to promote infection-associated EMT through activation of the p65/MMP-9 pathway in a *Mycobacterium bovis* infection/co-culture model. This finding highlights a potential link between RNA-binding proteins and EMT regulation, while its relevance to lung cancer CNS dissemination remains to be established [110].

The TME plays a pivotal role in determining the metastatic potential of cancer cells. The TME comprises various cell types, including stromal and immune cells, as well as ECM components, which can either support or inhibit tumor progression. Tumor-associated macrophages may adopt pro-tumorigenic roles by secreting growth factors and cytokines that promote metastasis [13,95,104]. Additionally, specific immune cell populations can create an immunosuppressive environment that facilitates tumor cell survival and dissemination [111].

Chemokines and their receptors are critical mediators of organ-specific metastasis. Tumor cells often express specific chemokine receptors that guide their migration to organs where corresponding chemokines are produced. Clinical and translational studies have reported elevated levels of CXCL12 and its receptor CXCR4 in patients with brain metastases compared with patients without metastatic disease [93]. This observation suggests that CXCL12/CXCR4 signaling may contribute to tumor cell proliferation and migration, potentially facilitating CNS dissemination. Experimental data further suggest that CX3CR1 expression may influence metastatic site preference, with CX3CR1-negative carcinomas showing a tendency toward brain dissemination and CX3CR1-expressing tumors preferentially spreading to other sites [112].

In addition to cytokines and markers of EMT loss, several growth factor pathways with protein kinase activity and their receptors have been implicated in the development of BM. Translational analyses of resected brain metastases across tumor types have shown increased EGFR/ERK pathway activation in CNS lesions, with lung cancer metastases displaying higher pathway expression than matched primary tumors [35].

Increased activation of c-MET and HIF signaling has been reported in brain metastases, including in EGFR-mutant NSCLC, suggesting a potential role in CNS adaptation and resistance to EGFR inhibition [113]. Additionally, the angiogenesis pathway plays a critical role in tumor invasion and CNS infiltration, with VEGF and its receptors promoting neo-vascularization and vascular permeability. Increased VEGF expression has been observed in NSCLC patients with brain involvement, especially those with adenocarcinoma. Preclinical studies indicate that silencing VEGF reduces the incidence of BM [114]. Moreover, studies have shown that monoclonal antibodies targeting VEGF receptors, such as bevacizumab, exhibit intracerebral activity due to the pathway’s overexpression in the brain [115].

The establishment of a pre-metastatic niche represents an important concept in metastatic dissemination. Extracellular vesicles, hypoxia, and extracellular matrix remodeling are well-established determinants of metastatic competence, although their specific contribution to lung cancer CNS colonization remains incompletely defined. For instance, tumor-secreted proteins can recruit immune cells and create an inflammatory environment that supports the survival of disseminated tumor cells [116]. Experimental evidence further suggests that niche formation is often mediated by exosomes and other extracellular vesicles that deliver signaling molecules to target organs [117].

Different organs exhibit distinct gene expression profiles that can influence the behavior of metastatic cells. Transcriptomic analyses of metastatic tissues have identified organ-specific genes that are upregulated in metastases, suggesting that the local microenvironment can induce specific transcriptional programs in tumor cells [118]. These observations support the broader concept that target organs can impose specific transcriptional states on metastatic cells; however, the extent to which these programs directly govern lung cancer brain metastasis or leptomeningeal dissemination remains incompletely established. Although these mechanisms are relevant to metastatic progression broadly, their direct and disease-specific role in lung cancer CNS colonization requires further validation.

Hypoxic conditions within the TME can significantly influence metastatic behavior. Mechanistic and preclinical studies have shown that tumors often adapt to low oxygen by activating HIF, which promotes angiogenesis and metabolic reprogramming [119]. These adaptations can enhance the survival and proliferation of metastatic cells in specific organs, such as the bone or liver, where hypoxic conditions may prevail [120].

The composition and remodeling of the ECM in target organs can also dictate metastatic behavior. Preclinical evidence suggests that the ECM provides structural support and biochemical signals that influence tumor cell migration and invasion. Specific ECM components, such as fibronectin and collagen, can facilitate the adhesion and growth of metastatic cells in the liver or lung [121]. Additionally, mechanistic preclinical studies have demonstrated that the interaction between tumor cells and the ECM can trigger signaling pathways that promote EMT, thereby enhancing the invasive potential of cancer cells [122].

Several pathways may have more direct support in lung cancer brain metastasis and leptomeningeal disease. Genomic divergence has emerged as one of the lines of evidence supporting the biological distinctiveness of CNS metastases. Comparative analyses of patient-derived brain metastases have identified alterations, including changes in the PI3K/AKT/mTOR axis, that may be present in brain lesions but absent from primary or extracranial sites [123]. Genomic profiling studies have further shown that CNS metastases in lung cancer exhibit increased tumor mutational burden, a greater fraction of the genome altered, and evidence of whole-genome duplication [28]. Although recurrent alterations in CDKN2A/B, TP53, EGFR, KRAS, and MYC have been documented across diverse histological subtypes of lung cancer [28,124,125,126], their specific roles in CNS tropism and metastatic progression remain poorly understood. Patient-based sequencing studies have also reported a higher frequency of CARD11 amplifications and a lower frequency of MDM2 amplifications in CNS metastases. Patients harboring atypical EGFR mutations or developing CNS involvement during osimertinib therapy have inferior survival outcomes. Whole-exome sequencing analyses identified recurrent amplifications of MYC, YAP1, and MMP13 in brain metastases, with subsequent functional studies supporting a role for these genes in promoting CNS metastatic dissemination [124]. Furthermore, comparative analyses suggest that the genomic landscapes of brain metastases and leptomeningeal metastases, as well as paired intra- and extracranial tumor samples, may be broadly similar. This observation implies that non-genomic mechanisms, including microenvironmental adaptation, cellular plasticity, and CNS-specific survival programs, may also be critical determinants of CNS colonization [127]. In summary, organ-specific metastasis reflects the convergence of general metastatic programs and mechanisms more specifically implicated in lung cancer CNS dissemination. EMT-associated plasticity, chemokine signaling, immune and stromal interactions, extracellular matrix remodeling, hypoxic adaptation, extracellular vesicle-mediated communication, and pre-metastatic niche formation provide a broad biological framework for organotropism. In lung cancer brain metastases and leptomeningeal disease, more disease-relevant evidence supports roles for oncogenic drivers such as EGFR and ALK, CXCL12/CXCR4 signaling, EGFR/ERK and c-MET/HIF pathway activation, VEGF-mediated vascular remodeling, CNS-enriched genomic alterations, and adaptive transcriptional programs. However, the strength of evidence varies across mechanisms, ranging from clinical and genomic observations to exploratory cohorts and preclinical models. Larger, clinically annotated studies are therefore needed to clarify which determinants are causal drivers of CNS tropism and which reflect broader features of metastatic competence.

## 4. Conclusions

The therapeutic landscape of NSCLC with CNS involvement has undergone a profound transformation over the past decade. Historically, brain metastases were associated with limited treatment options and poor outcomes, and management relied predominantly on local therapies. The development of CNS-active targeted therapies, antibody-based approaches, and improved molecular characterization has significantly expanded treatment options and altered the disease’s natural history for many patients. However, CNS metastases remain a biologically distinct disease compartment, shaped by unique microenvironmental, pharmacokinetic, and molecular factors that continue to hinder durable disease control. Moreover, many questions remain regarding the optimal integration of systemic therapies, surgery, and radiotherapy, as well as the best strategies for monitoring CNS disease and preserving long-term neurological function and quality of life. As survival continues to improve, treatment goals must increasingly extend beyond intracranial control to include neurocognitive outcomes, patient preferences, and overall quality of life.

Several key messages emerge from current evidence. Brain metastases should be viewed as a biologically distinct manifestation of NSCLC, marked by unique molecular, microenvironmental, and therapeutic challenges. The development of CNS-active targeted therapies has transformed outcomes in molecularly defined NSCLC, demonstrating that meaningful and durable intracranial disease control can be achieved in many patients. At the same time, optimal management increasingly requires a multidisciplinary, individualized approach that balances intracranial disease control, systemic efficacy, treatment-related toxicity, neurological function, and quality of life. Emerging technologies, including CSF-based liquid biopsy and advanced molecular profiling, are improving our understanding of CNS disease biology and may further refine disease characterization, monitoring, and treatment selection. Nevertheless, prospective studies remain essential to define the optimal sequencing and integration of systemic therapies, surgery, and radiotherapy in an era of increasingly effective CNS-penetrant treatments.

Ultimately, continued advances in CNS biology, molecular profiling, and therapeutic development offer an opportunity to further improve outcomes for patients with NSCLC and CNS involvement, moving beyond traditional disease control toward a more personalized, patient-centered model of care.

## Data Availability

No new datasets were generated or analyzed during the current study. This article is based on previously published literature, which is cited throughout the manuscript.
